# Online exams buffer the anxiety-procrastination-achievement pathway for distance learners with disabilities

**DOI:** 10.3389/fpsyg.2026.1806190

**Published:** 2026-05-15

**Authors:** Mesut Aydemir

**Affiliations:** Distance Education Department, Open Education Faculty, Anadolu University, Eskişehir, Türkiye

**Keywords:** academic procrastination, digital assessment, inclusive education, learners with disabilities, test anxiety

## Abstract

**Introduction:**

While Open and Distance Learning (ODL) expands access, the extent to which assessment modality is associated with psychological barriers for learners with disabilities remains underexplored. Aligned with the United Nations Sustainable Development Goal 4 (SDG 4)—ensuring inclusive and equitable quality education for all—this study contributes evidence on whether assessment design in open and distance systems supports or hinders equitable participation by learners with disabilities. Situated in debates on inclusive and equitable higher education, this study tests a self-regulation account in which test anxiety (TA) predicts academic procrastination (AP), which in turn predicts Grade Point Average (GPA), and examines whether exam environment (EE) moderates these psychological pathways differently for students with and without formally recognized disabilities.

**Methods:**

Data were collected via an online questionnaire from 667 undergraduates (118 learners with formally recognized disabilities) in a large-scale distance education system; GPA was obtained from institutional records. Structural equation modelling (SEM) and conditional process (moderated mediation) analyses were employed to test the hypothesized model.

**Results:**

AP mediates the negative association between TA and GPA for both disabled and non-disabled learners. For learners with disabilities, EE moderated the procrastination–GPA association: the negative association was stronger in face-to-face settings and weaker in online settings.

**Discussion:**

Given the cross-sectional design and self-selection into exam mode, these findings should be interpreted as associations rather than causal effects. Nonetheless, they suggest that carefully designed online assessment may function as an EE that helps reduce environmental frictions that can exacerbate the anxiety–procrastination cycle for some disabled learners, with implications for assessment practice (as a contextual factor) and accessibility and fairness (as contextual concerns) in open and distance higher education.

## Introduction

The democratization of higher education through Open and Distance Learning (ODL) relies heavily on the promise of “barrier-free” access. However, a critical tension remains in the “iron triangle” of ODL (access, cost, and quality): while content delivery has become increasingly flexible, assessment practice (as a contextual factor) often remains rigid. For learners with disabilities, this creates a paradox where the flexibility of learning materials is negated by the structural friction of traditional, high-stakes assessment environments. This study argues that to fully realize the inclusive potential of ODL, we must examine not only how students learn, but also how assessment is designed and delivered - particularly whether exams are administered online or face to face. Assessment conditions can function as situational cues that shape learners’ perceived control, stress responses, and self-regulation demands—factors that are known to influence procrastination and achievement outcomes in academic settings (Svartdal et al., 2020). This study is aligned with the United Nations Sustainable Development Goal 4 (SDG 4), which calls on nations to “ensure inclusive and equitable quality education and promote lifelong learning opportunities for all” by 2030 (United Nations, 2015). Target 4.5 specifically addresses the elimination of disparities for persons with disabilities in education. By investigating whether assessment modality is associated with psychological barriers that may differentially affect learners with disabilities, this study contributes evidence relevant to achieving SDG 4 commitments in open and distance higher education.

ODL refers to an educational approach that uses technology-mediated instruction to provide flexible access to higher education, typically characterized by asynchronous course delivery through pre-recorded lectures, digital textbooks, and online learning management systems, with minimal or no synchronous (real-time) interaction requirements. ODL in higher education widens access and inclusivity for varied learners by using technology to move course materials outside the physical classroom. For learners with disabilities, these programs bypass some of the physical and logistical obstacles found in face-to-face study (Mutanga, 2017). Recent comprehensive reviews confirm that despite progress, accessibility and inclusion in online higher education remain uneven, with persistent gaps in how digital platforms accommodate diverse learner needs (Lomellini et al., 2025). Yet, online learners often have fewer on-campus contacts and rapid help, but learners with disabilities also face other obstacles, such as inconvenient access paths, anxiety of asking questions, and self-control (Seale, 2013; Seale, 2022). Such strains can spark procrastination, boost exam nerves, and lower course grades. Still, evidence of how these psychological dynamics affect fully inclusive ODL contexts is few. In other words, digital expansion alone does not automatically translate into substantive participation or success for disabled students, raising critical questions about the social justice implications of ODL design. Providing computers and software will not eliminate internal obstacles like exam anxiety and delay-driven study practices (Broadbent and Poon, 2015).

While more studies now track the academic progress of learners with disabilities in ODL, few explore the psychological processes that help or hinder their success. Test anxiety (TA) is consistently associated with academic achievement and can be embedded in reciprocal dynamics with educational outcomes, highlighting its relevance as a central psychological barrier to performance (Steinmayr et al., 2016). ODL can remove physical barriers but does not fully address ongoing psychosocial challenges (Kotera et al., 2021). Understanding how these factors relate to performance metrics such as GPA could improve both course design and institutional policies. At the same time, such analyses contribute to broader interdisciplinary conversations in the social and behavioral sciences about how emotions, self-regulation, and contextual conditions intersect to shape stratified educational outcomes.

Moreover, the role of exam environments (EEs), whether online or face-to-face, emerges as a critical moderator that can either alleviate or exacerbate these psychological stressors. Despite being heralded as barrier-free, many EEs fail to fully accommodate cognitive, emotional, and sensory needs—especially in high-stakes assessments. Assessment design is therefore not only a pedagogical issue but also a contextual and policy-relevant one, with implications for how higher education systems operationalize commitments to inclusion and non-discrimination. Against this backdrop, the current study aims to investigate:

1. whether TA influences AP and GPA differently among learners with and without disabilities;
2. whether AP serves as a mediating variable between TA and GPA differently among learners with and without disabilities; and
3. whether EEs moderate these relationships differently among learners with and without disabilities.

By situating these questions in a large open and distance education system, the study speaks to international debates on accessibility and fairness (as contextual concerns), disability, and the governance of assessment in mass higher education.

## Theoretical background

### Educational Psychology foundations of the proposed model

**Figure 1:**
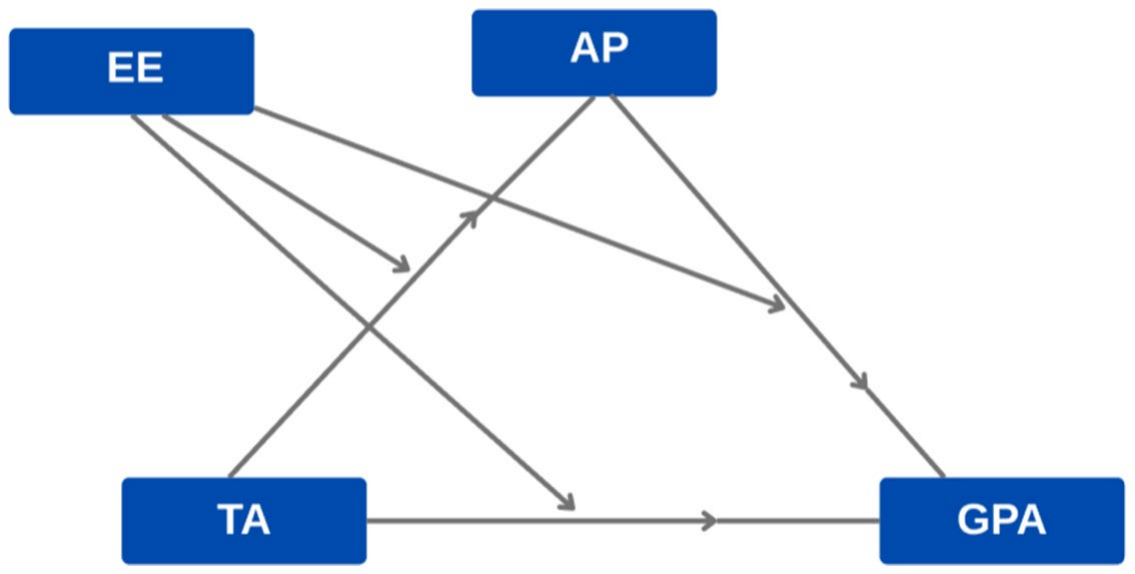
Proposed theoretical model.

The proposed model (Figure 1) is grounded in three converging theoretical frameworks from educational psychology. First, control-value theory (Pekrun, 2006, 2024) explains how perceived control over academic outcomes and subjective value of achievement shape emotional responses, including TA, which in turn influence cognitive and motivational processes. Second, self-regulation theory (Zimmerman, 2000; Steel, 2007) posits that AP represents a failure in self-regulatory processes—specifically, the inability to override short-term avoidance impulses in favor of long-term academic goals. When anxiety depletes self-regulatory resources, procrastination becomes a maladaptive coping strategy. Third, cognitive load theory (Sweller, 1988) suggests that anxiety-related worry consumes working memory capacity, reducing resources available for both task engagement (leading to procrastination) and test performance (directly impairing GPA). The exam environment enters the model as a contextual moderator that alters the situational demands and perceived control experienced by learners, consistent with Pekrun’s emphasis on environmental appraisals.

### Disability and educational access in ODL contexts

The ODL modality is often praised for its potential to serve as a “barrier-free” education system. Accessibility for learners with disabilities requires Universal Design for Learning (UDL) principles, which provide adaptive learning environments that meet diverse needs. UDL prompts educators to think beyond traditional accommodations and create inclusive practices from the ground up.

However, structural accessibility alone does not guarantee meaningful academic participation for students with disabilities. Access to technology must also consider usability, relevance, and alignment with educational goals. Learners often report that available tools do not meet their specific needs (Seale, 2022). Thus, digital materials should undergo ongoing testing and include student feedback to enhance accessibility. Horlin et al. (2024) stress that instead of simply accommodating individuals, course designers should use multiple modes to present content and engage students more fully. In ODL, the use of these principles is essential to guarantee that all learners, including those with disabilities, can access resources and participate in meaningful discourse.

It is important to acknowledge that “disability” encompasses a wide range of conditions—physical, sensory, neurological, psychological, and chronic health-related—each of which may interact with the anxiety–procrastination–achievement pathway through different mechanisms. For example, learners with attention-deficit/hyperactivity disorder (ADHD) or learning disabilities may face distinct self-regulation challenges (Sarid and Lipka, 2023), while those with sensory impairments may encounter specific accessibility barriers in EEs (Dahlstrom-Hakki and Alstad, 2019). Neurodivergent learners may benefit differentially from flexible assessment formats (Horlin et al., 2024). Despite this heterogeneity, the present study examines the shared experience of holding “formally recognized disability status” in the context of ODL assessment, with the limitation that disability-specific mechanisms are not disentangled (see Limitations).

Moreover, in ODL contexts, the lack of conventional support systems can heighten feelings of isolation and alienation among students, underscoring the necessity of promoting school connectivity even in virtual environments (Page et al., 2021). Studies have demonstrated that learners with disabilities face barriers in online learning frameworks due to inadequately designed instructional materials and lack of support (Finnerty et al., 2019; Olson et al., 2016). These barriers compound issues of equity and the need for robust technological infrastructures to support diverse learner needs (Park et al., 2019).

The principle of reasonable adjustments should be embedded in online-distance-learning systems to ensure inclusive education is applied in practice, not just in policy. This involves offering technical support, training educators in inclusive practices, and regularly assessing instructional designs to meet diverse learner needs (Raghunath et al., 2016; Seale, 2022). Addressing these areas helps create an environment where learners with disabilities can succeed. This study, therefore, compares both groups in the ODL context using TA, AP, GPA, and EE variables.

### Test anxiety: a cross-disability perspective

TA is a psychologically debilitating condition involving persistent worry and nervousness during assessments (Panayides et al., 2024). It includes cognitive concern, emotional distress, and heightened physical arousal (Zeidner, 2005). Studies show that learners with disabilities often face increased anxiety during tests, which can harm academic performance (Dahlstrom-Hakki and Alstad, 2019; Horlin et al., 2024). Those with invisible disabilities—such as ADHD, dyslexia, or generalized anxiety disorder—report especially high anxiety in traditional exam formats (Lombardi et al., 2011). Evidence also emphasizes that students with learning disabilities or ADHD experience emotional challenges (including anxiety and frustration) as they adapt to higher education demands, reinforcing the importance of examining anxiety pathways in disability-identified learner groups (Sarid and Lipka, 2023).

In ODL settings, isolation and delayed feedback can increase uncertainty, making anxiety a key barrier to performance. TA is also tied to cognitive overload and poor metacognitive regulation, which weaken independent learning and self-monitoring (Mutanga, 2017). Prior studies suggest that interventions targeting cognitive appraisals and academic self-efficacy can reduce both TA and subsequent AP, supporting the conceptual link between anxiety-driven avoidance and delayed task engagement (Krispenz et al., 2019). From the perspective of control-value theory (Pekrun, 2024), TA arises when learners appraise evaluative situations as important (high value) but perceive limited control over outcomes. In ODL contexts, the combination of high-stakes assessment with reduced support and feedback may heighten such appraisals, particularly for learners with disabilities. Moreover, evidence suggests that anxiety-related cognitive overload can directly disrupt information processing and recall, contributing to lower GPA (Cassady and Johnson, 2002). Therefore, this study posited the following hypotheses:

H_1a_: TA has a positive effect on AP for disabled groups.

H_1b_: TA has a positive effect on AP for non-disabled groups.

H_2a_: TA has a negative effect on GPA for disabled groups.

H_2b_: TA has a negative effect on GPA for non-disabled groups.

The paired hypothesis structure (a/b) reflects the study’s comparative design: the central research question is whether these pathways hold similarly or differ between groups, which requires formally stating and testing the same directional prediction in each subsample. The predicted directions are consistent across groups—positive for TA → AP, negative for TA → GPA—based on the convergent evidence reviewed above.

### Academic procrastination as a mediator

AP is closely tied to poor time management, low academic self-belief, and emotional-avoidance coping strategies (Steel, 2007). Cook and Ogden (2021) note that systemic barriers in education can lead to avoidance behaviors like procrastination among learners with disabilities. Grunschel et al. (2013) add that anxiety-driven procrastination is often a way to cope with stress, creating a cycle that heightens anxiety and weakens performance. A recent meta-analysis confirms that both active and passive procrastination are significantly related to academic performance, with passive (avoidant) procrastination showing consistently negative effects (Kooren et al., 2024). Importantly, Mastrokoukou et al. (2025) demonstrated that the drivers and consequences of AP differ between students with and without specific learning disabilities, supporting the need for group-specific modeling as conducted in the present study. Furthermore, Ye et al. (2025) showed that anxiety and self-control sequentially mediate the relationship between basic psychological needs and AP, reinforcing the self-regulation framework adopted here. Emerging research confirms that procrastination mediates the impact of TA on academic outcomes; learners who delay tasks due to anxiety perform worse than those who start early (Grunschel et al., 2013). Thus, procrastination is both a symptom and a driver of anxiety-related academic decline. In technology-mediated learning environments, behavioral indicators of procrastination have also been shown to predict academic performance, reinforcing the relevance of procrastination as a mechanism linking psychological strain to achievement outcomes (Cerezo et al., 2017). Hence, this study aimed to examine the following hypotheses:

H_3a_: AP has a negative effect on GPA for disabled groups.

H_3b_: AP has a negative effect on GPA for non-disabled groups.

H_4a_: AP mediates the relationship between TA and GPA for disabled groups.

H_4b_: AP mediates the relationship between TA and GPA for non-disabled groups.

### Exam environment as a moderator

Many learners view online exams as less stressful due to their flexibility. Consistent with this view, previous studies have shown that assessment format and online proctoring conditions can influence students’ test anxiety and performance (Núñez-Peña and Bono, 2020; Woldeab and Brothen, 2019). Another research comparing online and traditional student groups has reported meaningful differences in exam-related anxieties across learning modalities, underscoring that assessment context can alter the emotional experience of evaluation (Frey-Clark et al., 2019). Butler-Henderson and Crawford (2020) note that students generally prefer digital tests, pointing to a need for rethinking current assessment practices. However, online exams can still cause anxiety, as issues like software crashes or taking the test alone can increase stress and reduce confidence (Jiang et al., 2023).

An important consideration in the online exam context is technostress—the stress experienced by individuals in response to the use of information and communication technologies. In educational settings, technostress during online exams may arise from concerns about software malfunctions, internet connectivity issues, unfamiliarity with digital platforms, and the absence of immediate technical support (Saleem et al., 2024). For learners with disabilities, technostress may compound existing accessibility challenges, particularly when assistive technologies are incompatible with exam platforms or when digital interfaces lack adequate accessibility features. Thus, while online exams may reduce physical and logistical stressors associated with face-to-face testing, they may simultaneously introduce technology-related sources of anxiety, creating a nuanced trade-off that this study’s moderation analysis seeks to capture.

The mode of assessment affects learner engagement and emotional connection to academic work. While digital spaces may reduce anxiety for some, they can leave others—especially learners with disabilities—feeling isolated and disconnected from support networks. Creating an inclusive and supportive online environment is essential, as it impacts engagement, emotional health, and overall achievement (Page et al., 2021).

Research indicates that students with disabilities who feel connected and supported are more likely to perform well, even in the face of anxiety (Page et al., 2021). Moreover, Griful-Freixenet et al. (2017) states that test accommodations and inclusive assessment designs can significantly mediate the effects of psychological stressors on performance. Thus, exam settings may not only influence direct academic outcomes but also the strength and direction of psychological relationships underpinning those outcomes. Hence the study is aimed to test the following hypothesis:

H_5a_: Exam environment significantly moderates the indirect relationship between TA and GPA through AP disabled groups.

H_5a1_: Exam environment significantly moderates the direct relationship between TA and AP disabled group.

H_5a2_: Exam environment significantly moderates the direct relationship between AP and GPA disabled group.

H_5b_: Exam environment significantly moderates the indirect relationship between TA and GPA through AP non-disabled groups.

H_5b1_: Exam environment significantly moderates the direct relationship between TA and AP non-disabled group.

H_5b2_: Exam environment significantly moderates the direct relationship between AP and GPA non-disabled group.

H_6a_: Exam environment significantly moderates the direct relationship between TA and GPA disabled groups.

H_6b_: Exam environment significantly moderates the direct relationship between TA and GPA non-disabled group.

Taken together, the strands of literature on disability and access in ODL, TA, AP, and EEs point to the need for integrative research that cuts across educational psychology, disability studies, and digital higher education. Rather than treating assessment merely as a technical procedure, this study conceptualizes it as a psychological context that can amplify or buffer learners’ anxiety, self-regulation demands, and performance conditions across differently positioned students. In doing so, it aligns with interdisciplinary work in psychological science—particularly educational and social psychology—examining how EEs and institutional practices shape stress, motivation, self-regulation, and perceived fairness, with downstream consequences for achievement and participation.

## Materials and methods

### Study design

This study utilized a relational survey methodology (Tabachnick and Fidell, 2013) to examine the relationships among TA, AP, and GPA, as well as to identify both direct and indirect impacts of these variables. The moderating effect of exam types on this association is also analyzed. Structural equation modeling (SEM) was employed to evaluate the proposed theoretical model. Figure 1 illustrates the theoretical model.

### Ethics statement

This study was conducted in accordance with the ethical principles of the Declaration of Helsinki and was approved by the Ethics Committee of Anadolu University (Approval Code: 820998). In line with the approved protocol, participants were informed about the aims and procedures of the study, the voluntary nature of their participation, and their right to withdraw at any time without penalty. Informed consent was obtained from all participants prior to data collection, in accordance with the requirements of the institutional review board.

### Participants

The study group comprised university students enrolled in the Open Education System (OES) in Türkiye during the Fall and Spring semesters of the 2024–2025 academic year. Anadolu University’s Open Education System is one of the world’s largest distance education systems, serving over 1 million active students across a wide range of undergraduate programs. Course content is delivered primarily through asynchronous formats: pre-recorded video lectures on a dedicated platform (Anadolum e-Campus), digital textbooks, and supplementary online materials. There are no mandatory synchronous (real-time) sessions. For assessment, students may choose between face-to-face exams (administered at designated exam centers across Türkiye under proctored, supervised conditions) and online exams (taken remotely via a web-based platform with identity verification at login but without continuous remote proctoring software such as lockdown browsers or AI-based monitoring). Both formats use multiple-choice questions. The online exam option was expanded following the COVID-19 pandemic and has been retained as a permanent alternative. Students self-select their preferred exam mode each semester. Data were obtained from 122 learners with disabilities and 550 learners without disabilities. Following the screening for missing data, as well as univariate and multivariate outliers, the analyses proceeded with 118 learners with disabilities and 549 learners without disabilities. Among the learners with disabilities, the mean number of reported disabilities was 1.77 (SD = 1.58), and the average disability severity rate was 54.76%.

The disability group was heterogeneous, encompassing musculoskeletal conditions (17.8%), mental/psychological/behavioral disorders (16.9%), nervous system conditions (15.3%), ear/nose/throat conditions (11.9%), cardiovascular conditions (8.5%), visual impairments (8.5%), internal medicine conditions (7.6%), oncological diseases (3.4%), endocrine conditions (2.5%), respiratory conditions (2.5%), hematopoietic conditions (1.7%), urogenital conditions (1.7%), and other categories (see Table 1 for full details). This heterogeneity is typical of ODL populations, where diverse learners self-select into flexible programs. While this diversity limits disability-specific inferences, it reflects the population that ODL institutions actually serve and allows the study to examine the shared experience of formally recognized disability status in the assessment context.

**Table 1:** Sociodemographic characteristics of the study group.

Baseline characteristic	Disabled		Non-disabled	
	*n*	*%*	*n*	*%*
Gender				
Female	51	43,2	349	63,6
Male	67	56,8	200	36,4
Type of disability				
CP patient	1	.8	—	—
Walks with support	1	.8	—	—
Endocrine system	3	2.5	—	—
Visual system	10	8.5	—	—
Hematopoietic system	2	1.7	—	—
Internal medicine	9	7.6	—	—
Cardiovascular system	10	8.5	—	—
Musculoskeletal system	21	17.8	—	—
Ear, nose, and throat system	14	11.9	—	—
Oncological diseases	4	3.4	—	—
Nervous system	18	15.3	—	—
Respiratory system	3	2.5	—	—
Urogenital system	2	1.7	—	—
Mental, psychological and behavioral disorders	20	16.9	—	—
Total	118	100.0	—	—

### Measures

*Personal Information Form*: The personal information form was developed by researcher and includes questions about students’ gender, age, program type, disability status (formally recognized disability with official documentation), disability type and severity, and preferred exam environment.

*Westside Test Anxiety Scale*: Originally created by Driscoll (2007), the 10-item, one-dimensional, five-point Likert-type scale (1 = never true to 5 = always true) was adapted into Turkish by Totan and Yavuz (2009). The scale is designed to assess impairment caused by TA, capturing cognitive worry, physiological arousal, and behavioral aspects within a single unidimensional construct. The exploratory factor analysis yielded a 46.05% explained variance. The confirmatory factor analysis’s results showed that the goodness of fit indices were within an acceptable range. The coefficient for Cronbach’s Alpha was determined to be 0.89. Sample items include: “I am so nervous during tests that I cannot remember facts I have learned”; “During tests, I find myself thinking of the consequences of failing”; and “After a test, I worry about whether I did well enough.”

*Academic Procrastination Scale-Short Form (APS-SF)*: The scale is a one-dimensional 5-item, 5-point Likert-type scale (1 = strongly disagree to 5 = strongly agree) that was first created by McCloskey (2011) and translated into Turkish by Balkıs and Duru (2022). The APS-SF measures the general tendency to delay academic tasks as a single unidimensional construct. The goodness of fit indices was determined to be at an acceptable level following the confirmatory factor analysis. The reliability coefficient for Cronbach’s Alpha was determined to be 0.88. Sample items include: “I delay making decisions until it’s too late”; “I put off projects until the last minute”; and “When I have a deadline, I wait till the last minute.”

### Statistical analysis

Data were collected from 550 non-disabled learners and 122 learners with disabilities. Analyses were performed using IBM SPSS Statistics (Version 26), Jamovi (Version 2.4.14), and the PROCESS macro. The dataset was first checked for excessive and missing values. Mahalanobis distances (*p* < 0.001) were used to detect multivariate outliers. No univariate outliers were found, but two multivariate outliers were removed. Final analyses included 549 non-disabled and 118 learners with disabilities.

The assumptions of normality, linearity, and multicollinearity were checked, with no issues found. Descriptive statistics and Pearson correlations gave an initial view of how the study variables relate. SEM was used to examine these relationships and model components. Both measurement and structural models were tested, and model validity was assessed using fit indices (Haryono, 2017). Statistical stability was evaluated through bootstrapping, based on 5,000 resamples and 95% confidence intervals (Preacher and Hayes, 2008).

A moderated mediation analysis was conducted using Jamovi and the PROCESS macro (Model 59) to examine how variables interact. GPA was the outcome variable, TA the independent variable, and AP the mediator. Moderated mediation models are used when a predictor’s indirect effect on an outcome depends on a moderator’s level. These models help explain both the mediating process and how effects change under different conditions (Hayes, 2022). The analysis was further supported by the jAMM: Jamovi Advanced Mediation Models module (Gallucci, 2020).

## Results

### Descriptive statistics and correlations

Descriptive statistics were computed for all variables, and Pearson correlation coefficients were employed to examine the relationships among TA, AP, and GPA among learners. Table 2 presents the means, standard deviations, and correlation coefficients for each construct.

**Table 2:** Descriptive statistics and correlations between study variables.

Group	Variables	M	SD	Skewness coefficient	Kurtosis coefficient	1	2	3
Disabled	1. TA	34.36	10.92	−0.265	−0.725	—		
	2. AP	12.22	4.69	0.394	−0.404	0.592*	—	
	3. GPA	1.72	1.05	−0.209	−0.999	−0.452*	−0.506*	—
Non-disabled	1. TA	31.92	9.74	0.235	−0.547	—		
	2. AP	12.70	5.43	0.52	−0.64	0.417*	—	
	3. GPA	2.11	1.03	−0.248	−0.784	−0.225*	−0.256*	—

M, mean; SD, standard deviation; TA, test anxiety; AP, academic procrastination; GPA, grade point average. *p < 0.05.

Significant associations among the constructs included in the model are presented in Table 2. While there was a significant positive relationship between TA and AP (r_TA-AP_ = 0.592 for learners with disabilities, r_TA-AP_ = 0.417, *p* < 0.05 for learners without disabilities), there were significant negative relationships between TA and AP and GPA (r_TA-GPA_ = −0.452, r_AP-GPA_ = −0.506, *p* < 0.05 for learners with disabilities; r_TA-GPA_ = −0.225, r_AP-GPA_ = −0.256, *p* < 0.05 for learners without disabilities).

Notably, mean TA levels were comparable across groups (disabled: M = 34.36, SD = 10.92; non-disabled: M = 31.92, SD = 9.74), with only a modest numerical difference. This pattern is consistent with prior research suggesting that TA is a widespread experience across student populations, regardless of disability status (Zeidner, 2005; Panayides et al., 2024). The critical question for this study is not whether disabled learners experience more anxiety, but whether the downstream consequences of equivalent anxiety levels differ—that is, whether the same levels of TA produce stronger procrastination and steeper GPA declines for learners with disabilities due to compounding contextual barriers.

### Findings regarding the theoretical model

The measurement model was examined first, as it is essential for evaluating the path and structural models. Item reliabilities were assessed to ensure indicators reflected their latent constructs, with all factor loadings above the 0.50 threshold (Chin, 2010). Reliability, convergent validity, and discriminant validity were also tested using Cronbach’s alpha, composite reliability (CR), and average variance extracted (AVE). As shown in Table 3, these indicators confirm the measurement model is suitable for further structural analysis.

**Table 3:** Reliability of measurement model.

Variables	Disabled			Non-disabled		
	Cα	CR	AVE	Cα	CR	AVE
TA	0.944	0.945	0.612	0.921	0.922	0.521
AP	0.838	0.844	0.525	0.900	0.900	0.645

The Cronbach’s alpha coefficients for the scale scores exceeded the recommended threshold of 0.70, indicating acceptable internal consistency and suggesting that the results are reliable (Creswell, 2012). Additionally, both the Average Variance Extracted (AVE) and Composite Reliability (CR) values surpassed the recommended benchmarks. According to Fornell and Larcker (1981), CR values should be at least 0.60 and AVE values should exceed 0.50. Taken together, these findings support the reliability of the scales, as well as the convergent and discriminant validity of the measurement constructs used in assessing the experiences of the learners.

Following the evaluation of the measurement model, the structural model was examined. SEM was employed to test the proposed theoretical framework. The analysis demonstrated that the model met the recommended thresholds for goodness-of-fit indices, indicating an adequate fit between the model and the data. Detailed model fit indices and corresponding results are presented in Table 4.

**Table 4:** Goodness of fit indices of the model.

Fit index	Disabled scores	Non-disabled scores	Good fit indices	Acceptable fit indices
*χ*^2^/df	2.06 (241/117)	4.37 (511/117)	≤2	≤5
RMSEA	0.09	0.08	≤0.05	≤0.10
SRMR	0.07	0.04	≤0.05	≤0.10
CFI	0.91	0.92	≥0.95	≥0.90
TLI	0.89	0.91	≥0.95	≥0.90
RNI	0.91	0.92	≥0.95	≥0.90

*χ*^2^/df, Relative chi-square; RMSEA, Root Mean Squared Error of Approximation; SRMR, Standardized Root Mean Square Residual; CFI, Comparative Fit Index; TLI, Tucker–Lewis Index; RNI, Relative Centrality Index (Hooper et al., 2008; Tabachnick and Fidell, 2013).

The model fit indices (Table 4) indicate acceptable but not optimal fit, particularly for the non-disabled group (*χ*^2^/df = 4.37, RMSEA = 0.08, TLI = 0.91). Several factors contribute to these values. First, the relatively high *χ*^2^/df for the non-disabled group is partly attributable to the larger sample size (*n* = 549), as chi-square statistics are sensitive to sample size and tend to inflate with larger samples even when model misspecification is minor (Hooper et al., 2008). Second, RMSEA values of 0.08–0.09 remain within the commonly accepted threshold of ≤0.10 for adequate fit (Browne and Cudeck, 1993), though they indicate room for model improvement. Third, TLI = 0.89 for the disabled group is marginally below the 0.90 threshold, likely reflecting the smaller subsample (*n* = 118) and attendant estimation instability. Importantly, CFI values (≥0.91) and SRMR values (≤0.07) meet conventional benchmarks, and the overall pattern suggests that the model captures the primary structural relationships adequately despite not achieving excellent fit across all indices.

**Figure 2:**
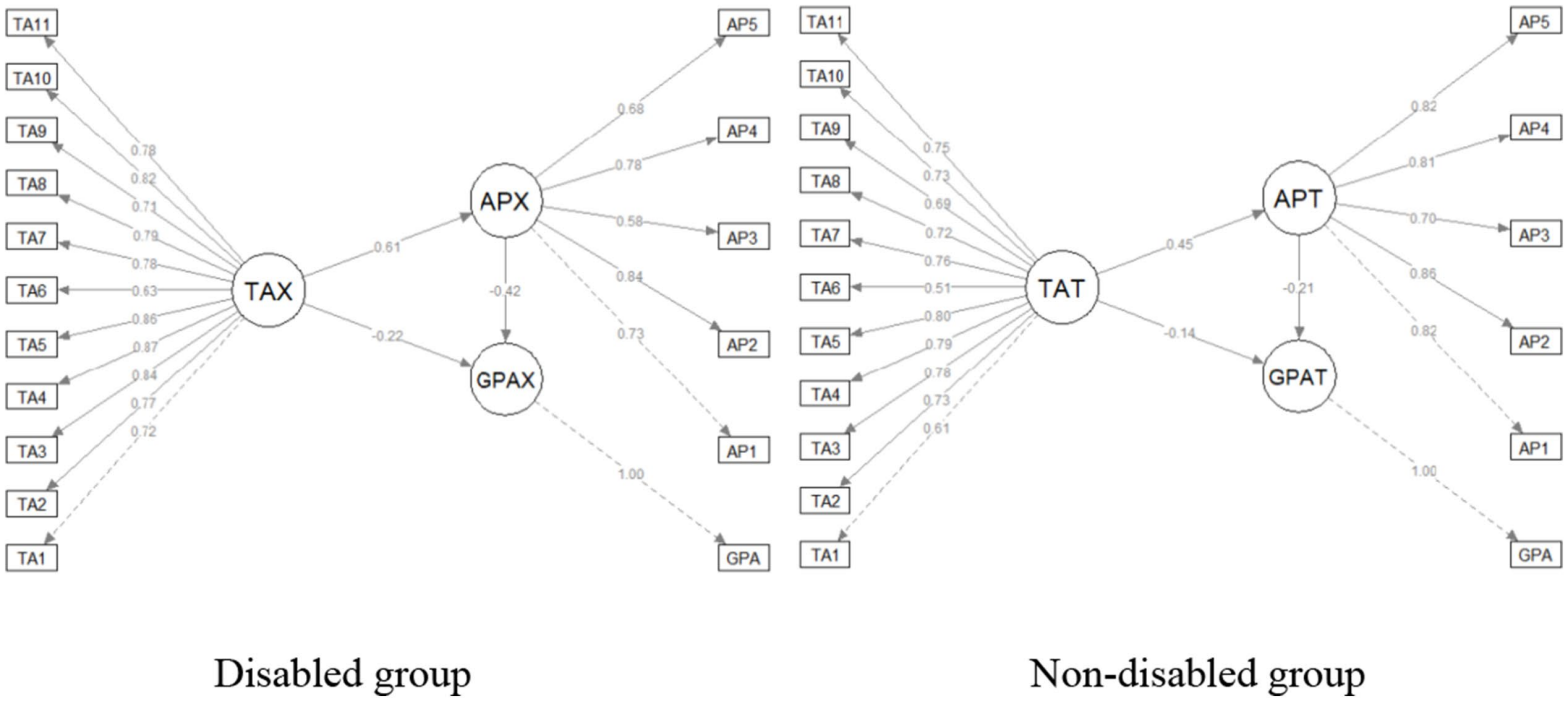
Results of the structural model.

Furthermore, all of the model’s fit indicators satisfied the required standards for these two groups when the goodness of fit statistics of the scale scores was analyzed. These results demonstrate the validity of the models. Table 5 and Figure 2 present the results of the path analysis carried out to ascertain the function of mediating variables.

**Table 5:** Results of path analysis on the mediation of AP.

Path coefficients and indirect effect	Group	*Estimate*	SE	*Beta*	*t*-value	*p*	CI
TA ⇒ AP	Disabled	0.634	0.159	0.606	3.99	<0.001	[0.306, 0.931]
	Non-disabled	0.661	0.076	0.451	8.71	<0.001	[0.514, 0.814]
TA ⇒ GPA	Disabled	−0.262	0.128	−0.220	−2.04	0.041	[−0.507, −0.006]
	Non-disabled	−0.197	0.070	−0.137	−2.80	0.005	[−0.333, −0.058]
AP ⇒ GPA	Disabled	−0.473	0.132	−0.415	−3.58	<0.001	[−0.727, −0.206]
	Non-disabled	−0.203	0.049	−0.206	−4.09	<0.001	[−0.300, −0.103]
TA ⇒ AP ⇒ GPA	Disabled	−0.300	0.110	−0.252	−2.716	0.007	[−0.573, −0.127]
	Non-disabled	−0.134	0.035	−0.093	−3.834	<0.001	[−0.210, −0.072]

TA, test anxiety; AP, academic procrastination; GPA, grade point average; SE, standard error; CI = 95% bias-corrected bootstrap confidence interval.

Consistent with H_1a_–H_3b_, TA was positively associated with AP (disabled: *β* = 0.606, *p* < 0.001; non-disabled: *β* = 0.451, *p* < 0.001) and AP was negatively associated with GPA (disabled: *β* = −0.415, *p* < 0.001; non-disabled: *β* = −0.206, *p* < 0.001). TA also showed a significant direct negative association with GPA when AP was included in the model (disabled: *β* = −0.220, *p* = 0.041; non-disabled: *β* = −0.137, *p* = 0.005). Bootstrapped indirect effects supported mediation in both groups (disabled: βindirect = −0.252, *p* = 0.007; non-disabled: βindirect = −0.093, *p* < 0.001), indicating that AP partially mediates the TA–GPA association.

As Table 5 shows, the bootstrapped confidence intervals (CIs) test findings validated the presence of indirect effects. Given that the bootstrap 95% CIs for the mediator AP do not contain zero, the results showed strong evidence for the presence of an indirect effect. Thus, evidence of mediation relationships was provided and H_4a_ and H_4b_ are supported.

The moderator effect of EE on the direct and indirect effects of TA in predicting GPA is given in Table 6. It is seen that the interaction of TA and EE has no significant effect on AP and GPA for disabled and non-disabled groups (*p* > 0.05). The interaction between AP and EE is significant on GPA (*p* < 0.05) for disabled groups, while the interaction between AP and EE is insignificant for non-disabled groups (*p* > 0.05). In conclusion, the effect of AP on GPA was moderated by EE for disabled groups.

**Table 6:** Moderated mediation analysis.

Interaction		Estimate	*SE*	*β*	z	*p*
TAxEE ⇒ AP	Disabled	0.056	0.078	0.053	0.715	0.475
	Non-disabled	−0.012	0.049	−0.011	−0.251	0.802
TAxEE ⇒ GPA	Disabled	−0.006	0.020	−0.024	−0.287	0.774
	Non-disabled	0.005	0.011	0.022	0.437	0.662
EExAP ⇒ GPA	Disabled	0.096	0.044	0.534	2.166	0.030
	Non-disabled	−0.014	0.017	−0.086	−0.807	0.420

TA, test anxiety; AP, academic procrastination; GPA, grade point average; EE, exam environment; SE, standard error.

**Figure 3:**
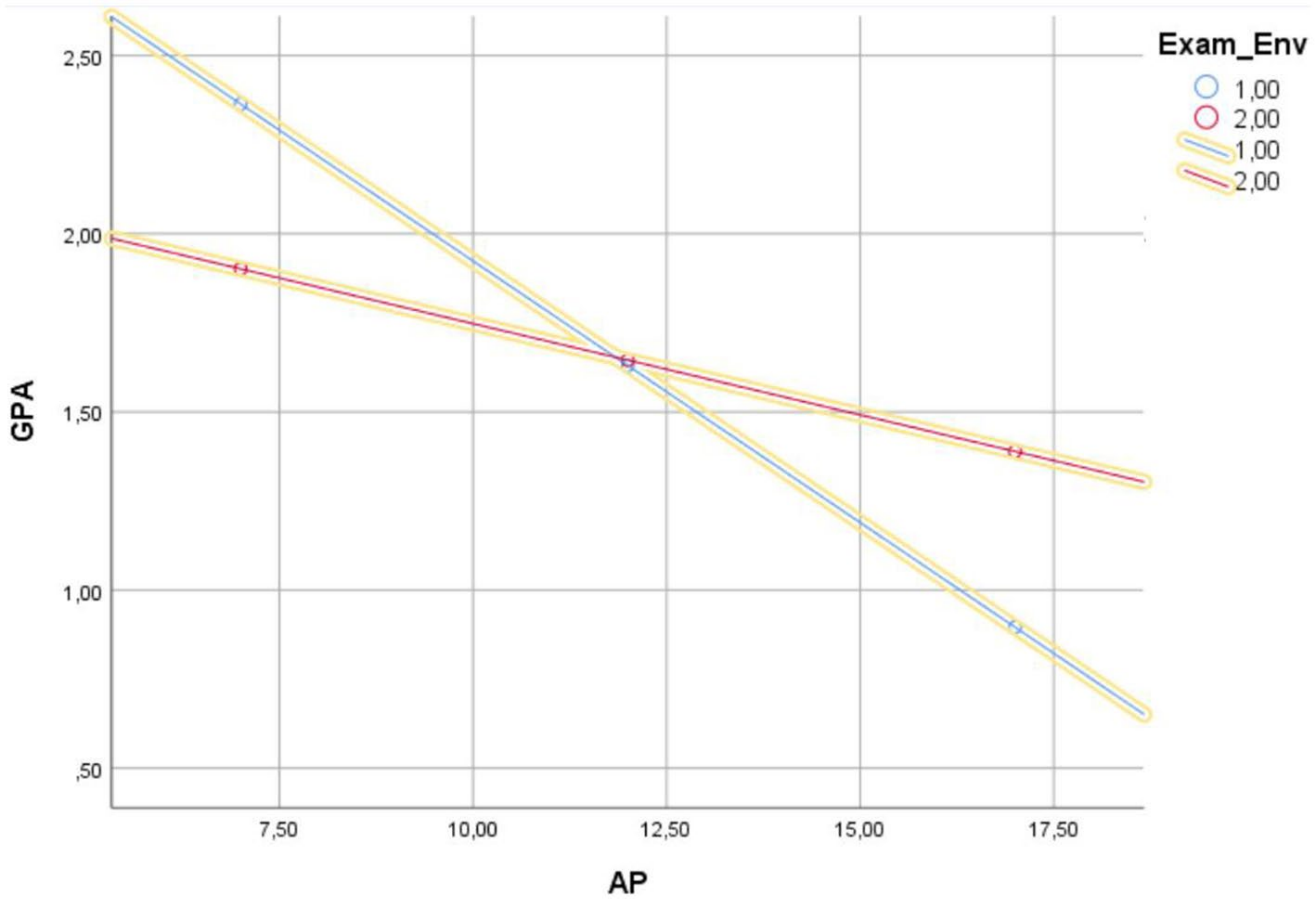
Exam type as a moderator of the relationship between AP and GPA.

The moderated mediation model 59 showed that the conditional indirect effect on GPA was due to TA through AP, and this effect varied by exam environment for the disabled group. The main effect of AP on GPA was significant for both groups. While there was a moderating effect of exam environment on the direct effect of AP on GPA for the disabled group (*β* = 0.534, *p* < 0.05), there was no moderating effect of exam environment for the non-disabled group (*p* > 0.05). When Table 7 and Figure 3 are examined, it is seen that the face-to-face exam environment (*β* = −0.591, CI [−0.238, −0.091], *p* < 0.05) has a higher direct effect of AP on GPA than the online exam environment (*β* = −0.233, CI [−0.104, −0.003], *p* < 0.05) in the disabled group. Therefore, higher AP level was associated with a lower GPA level.

**Table 7:** Results of path analysis on the moderation of exam environment for disabled group.

Group	Type	Effect	Estimates	SE	Beta	*z*	*p*	CI (%95)
Face-to-face component		TA ⇒ AP	0.149	0.060	0.347	2.46	0.014	[0.016, 0.276]
		AP ⇒ GPA	−0.147	0.022	−0.591	−6.66	<0.001	[−0.238,-0.091]
	Indirect	TA ⇒ AP ⇒ GPA	−0.022	0.009	−0.205	−2.31	0.021	[−0.054, −0.003]
	Direct	TA ⇒ GPA	−0.016	0.015	−0.156	−1.12	0.264	[−0.050, 0.024]
	Total	TA ⇒ GPA	−0.038	0.016	−0.400	−2.47	0.014	[−0.071, −0.001]
Online component		TA ⇒ AP	0.204	0.049	0.476	4.18	<0.001	[0.102, 0.317]
		AP ⇒ GPA	−0.051	0.022	−0.233	−2.32	0.020	[−0.104, −0.003]
	Indirect	TA ⇒ AP ⇒ GPA	−0.010	0.005	−0.111	−2.03	0.042	[−0.024, −0.001]
	Direct	TA ⇒ GPA	−0.022	0.013	−0.237	−1.79	0.074	[−0.044, 0.002]
	Total	TA ⇒ GPA	−0.033	0.013	−0.342	−2.61	0.009	[−0.055, −0.013]

TA, test anxiety; AP, academic procrastination; GPA, grade point average; SE, standard error; CI, 95% confidence interval.

As seen in Figure 3, higher levels of AP are significantly associated with lower levels of GPA in face-to-face exam environment compared to the online exam environment. Those who have high AP and prefer face-to-face exam environment tend to have more GPA declines than those who choose online exams. Furthermore, the relationship between AP and success is stronger for those who choose face-to-face exam environment. In conclusion, H_5a_ and H_5a2_ are supported while H_5a1_, H_5b_, H_5b1_, H_5b2_, H_6a_ and H_6b_ are not supported.

The 95% CIs of the bootstrap approach were used to analyze indirect effects at two levels of the test environment in order to assess the conditional indirect effects of the TA level on GPA via AP. The results demonstrated that the conditional indirect effect of TA on GPA through AP was significant when test environment was added to the model as a moderator variable, and 95% CIs for both groups did not contain 0 (*β* = −0.205 for face-to-face group, *β* = −0.111 for online group, *p* < 0.05). The indirect negative effect between TA and GPA via AP was stronger for students who chose face-to-face exam type. Both graphical representation and statistical findings support H_5a_, and H_5a2_.

## Discussion

This study contributes to wider social-science debates about the consequences of digitalization for equity in higher education. By combining psychological constructs (TA and AP) with structural features of assessment design (EE) in a large ODL system, the study offers an interdisciplinary account of how digital infrastructures can both alleviate and reproduce disadvantage for learners with disabilities.

### The anxiety-procrastination cycle in ODL

The primary aim of this study was to dismantle the structural relationships between TA, AP, and achievement. In doing so, we sought to move beyond individual “deficit” explanations and to locate anxiety and procrastination within the institutional conditions of assessment in ODL. This interpretation aligns with research by Svartdal et al. (2020) arguing that procrastination is not only an individual trait but is also fostered by study environments and contextual factors that increase distraction, uncertainty, and self-regulatory load. Consistent with the proposed theoretical model, the findings confirm a universal mechanism of “anxiety-driven avoidance” across both groups. We observed that TA not only directly impairs GPA but significantly fuels AP, which subsequently degrades academic performance. This aligns with self-regulation theory, which posits that learners experiencing high cognitive concern (anxiety) often resort to task delays as a maladaptive emotional coping strategy. The confirmation of AP as a partial mediator (H_4a_/H_4b_) suggests that the decline in grades is not solely due to the cognitive interference of anxiety during the test, but also due to the cumulative loss of preparation time caused by antecedent procrastination. This finding reinforces that even in flexible ODL environments, the psychological pressure of assessment disrupts self-regulatory processes for all students.

### The disproportionate burden on learners with disabilities

While the anxiety-procrastination mechanism exists for all learners, the SEM revealed that these pathways are significantly more detrimental for learners with disabilities. The path coefficients linking anxiety to procrastination and procrastination to GPA were markedly stronger in the disabled group. This disparity corroborates earlier research suggesting that learners with disabilities in ODL contexts face a “double burden”: they must navigate not only the standard academic rigor but also specific psychosocial and accessibility barriers that exacerbate cognitive load. For these learners, procrastination is likely less a matter of time management and more a symptom of executive functioning fatigue or fear of confronting inaccessible materials. Consequently, the “cost” of procrastination is higher; a non-disabled student might recover from a delay, whereas for a student with disabilities, the compounded stress creates a steeper decline in GPA.

### Assessment modality as a technological equalizer

The most critical contribution of this study is that the exam environment—face-to-face versus online assessment—moderates the anxiety–procrastination–achievement pathway for learners with disabilities. Specifically, among disabled learners the procrastination–GPA association was stronger in face-to-face exams and weaker in online exams, whereas no comparable moderation emerged for non-disabled learners. Because exam environment was self-selected and the data are cross-sectional, this pattern should not be interpreted as definitive causal evidence; unobserved confounders (e.g., prior achievement, digital access, or disability characteristics) may influence both exam-mode choice and outcomes. Nevertheless, the results are consistent with a “buffer” account in which online assessment may reduce situational frictions (e.g., travel, sensory overload, and rigid time–pressure cues) that can intensify anxiety-related avoidance for some disabled students.

## Conclusion

This study shows that TA significantly increases AP and directly lowers GPA in both learners with disabilities and those without. AP partly mediated the link between TA and achievement in both groups. These findings suggest that higher TA leads to more AP, which then harms academic performance for all learners.

The test environment (face-to-face vs. online) significantly moderated the mediated pathway between TA and GPA through AP only for learners with disabilities. For these learners, procrastination had a stronger negative effect on achievement in face-to-face exams, though the effect remained negative in both formats. In contrast, the test environment did not significantly moderate any relationships for learners without disabilities.

This study adds to the literature by comparing how TA affects AP and achievement in learners with and without disabilities. The stronger impact on learners with disabilities highlights the need for targeted support to manage exam-related anxiety in ODL. While ODL may reduce physical barriers, it does not fully address psychological and self-regulation challenges (Seale, 2013; Broadbent and Poon, 2015). The findings stress the importance of including psychosocial support alongside physical accessibility in inclusive ODL design.

The findings also imply that AP’s association with achievement is shaped by institutional and environmental conditions, particularly for students with disabilities. Accordingly, ODL providers may consider support systems that strengthen time management and self-regulation (e.g., structured feedback, formative assessments, and regular low-stakes assignments), and evaluate their effectiveness for different disability profiles.

While ODL can reduce physical barriers, limited self-regulation supports and psychosocial scaffolding may contribute to anxiety, procrastination, and lower achievement—especially for learners with disabilities. These findings highlight the importance of pairing accessibility with evidence-informed self-regulation supports and anxiety-reduction resources, tailored to learners’ needs.

This study contributes to the literature by comparing the moderating role of the test environment for learners with and without disabilities. For learners with disabilities, the test environment affects indirect relationships between variables, highlighting the need for flexible, accessible, and psychologically supportive assessment models in ODL. In contrast, the absence of a significant moderating effect for learners without disabilities suggests that assessment improvements should focus on the specific needs of learners with disabilities to promote equity and inclusion in ODL.

### Practical implications, recommendations and limitations

One practical implication is that, among learners with disabilities, higher AP was more strongly associated with lower GPA in face-to-face examinations than in online examinations. This suggests that expanding access to accessible online exam options and considering flexible assessment arrangements (e.g., time allowances and choice of mode where feasible) may help mitigate disadvantage in large-scale distance education systems. These implications should be weighed alongside academic integrity requirements and local regulatory constraints.

Beyond accommodation checklists, learning management system (LMS)-enabled assessment design can be leveraged as part of broader accessibility and fairness (as contextual concerns) strategies. In our data, online exam settings were associated with a weaker procrastination–GPA link among learners with disabilities, suggesting potential benefits of offering accessible online options alongside face-to-face formats where integrity and feasibility requirements allow. Institutions might also consider reducing reliance on single high-stakes assessments by using LMS features (e.g., adaptive release) to scaffold learning through smaller, lower-stakes components, complemented by supportive digital “nudges” and progress feedback. For policymakers and quality-assurance bodies, criteria for “accessible assessment” may need to address not only individual accommodations but also the temporal structure, usability, and psychological demands of assessment ecosystems.

At the governance level, UDL can provide a guiding framework for assessment design in ODL systems. Rather than mandating a single exam mode, institutions may consider flexible models that allow multiple modes of assessment (including accessible online examinations) supported by clear integrity safeguards. Training for educators and exam staff on disability-inclusive assessment and the psychosocial consequences of exam settings may further help to reduce stressors and support autonomy for learners with disabilities.

Future research could expand on this study by examining similar dynamics across different disability groups (e.g., learners with hearing impairments, physical disabilities, or neurodivergence) and types of examinations. Longitudinal research may also offer valuable insights into how the relationships among test anxiety, procrastination, and achievement evolve over time.

This study has some limitations. First, data were collected during one academic term, limiting causal inference; future longitudinal and experimental or quasi-experimental designs would strengthen claims about directionality. Second, the test environment was classified only as online or face-to-face; future research should explore finer distinctions (e.g., proctored vs. non-proctored online exams, proctoring intensity, and accessibility features). Third, exam environment was self-selected rather than randomly assigned, so selection effects and unmeasured confounding may partly explain the observed moderation patterns. Fourth, the disability group was smaller and heterogeneous in type and severity, encompassing physical, sensory, neurological, psychological, and chronic health conditions that may interact with the anxiety–procrastination pathway through distinct mechanisms. The sample size for individual disability categories was too small for separate modeling, and future research should examine these dynamics across specific disability subgroups. Fifth, although separate reliability and validity indicators were adequate in both groups (Table 3), we did not conduct formal measurement invariance (configural, metric, scalar) testing across the disabled and non-disabled groups. Without measurement invariance evidence, it is possible that items function differently across groups, and between-group comparisons of structural coefficients should be interpreted with caution. Future studies with larger disability subsamples should conduct multi-group confirmatory factor analysis to establish measurement equivalence before comparing path coefficients (Farmer et al., 2024). Sixth, all data were collected from a single large-scale OES in Türkiye (Anadolu University). Although this system is among the world’s largest ODL providers, findings may not generalize to dissimilar higher education contexts with different institutional structures, assessment policies, disability support services, or cultural norms. Lastly, while GPA came from official records, TA and AP were self-reported, which may introduce common-method or reporting biases. Comparative and cross-jurisdictional research across different ODL systems and regulatory contexts would help clarify how far the patterns identified here generalize across national settings and models of digital assessment.

## Data Availability

The raw data supporting the conclusions of this article will be made available by the authors, without undue reservation.

## References

[ref1] Balkıs M. Duru E. (2022). Akademik Erteleme Ölçeği’nin Kısa Formunun Psikometrik Özelliklerinin İncelenmesi. Pamukkale Üniversitesi Eğitim Fakültesi Dergisi 54, 410–425. doi: 10.9779/pauefd.952291

[ref2] Broadbent J. Poon W. L. (2015). Self-regulated learning strategies & academic achievement in online higher education learning environments: a systematic review. Internet High. Educ. 27, 1–13. doi: 10.1016/j.iheduc.2015.04.007

[ref3] Browne M. W. Cudeck R. (1993). “Alternative ways of assessing model fit,” in Testing Structural Equation Models, eds. Bollen K. A. Long J. S. (Newbury Park: Sage), 136–162.

[ref4] Butler-Henderson K. Crawford J. (2020). A systematic review of online examinations: a pedagogical innovation for scalable authentication and integrity. Comput. Educ. 159:104024. doi: 10.1016/j.compedu.2020.104024, 32982023 PMC7508171

[ref5] Cassady J. C. Johnson R. E. (2002). Cognitive test anxiety and academic performance. Contemp. Educ. Psychol. 27, 270–295. doi: 10.1006/ceps.2001.1094

[ref6] Cerezo R. Esteban M. Sánchez-Santillán M. Núñez J. C. (2017). Procrastinating behavior in computer-based learning environments to predict performance: a case study in Moodle. Front. Psychol. 8:1403. doi: 10.3389/fpsyg.2017.01403, 28883801 PMC5573842

[ref7] Chin W. W. (2010). “How to write up and report PLS analyses,” in Handbook of Partial Least Squares. Springer Handbooks of Computational Statistics, eds. Esposito Vinzi V. Chin W. Henseler J. Wang H. (Berlin: Springer), 655–690.

[ref8] Cook A. Ogden J. (2021). Challenges, strategies and self-efficacy of teachers supporting autistic pupils in contrasting school settings: a qualitative study. Eur. J. Spec. Needs Educ. 37, 371–385. doi: 10.1080/08856257.2021.1878659

[ref9] Creswell J. W. (2012). Educational Research: Planning, Conducting, and Evaluating Quantitative and Qualitative Research. 4th Edn New York: Pearson Education Inc.

[ref10] Dahlstrom-Hakki I. Alstad Z. (2019). Challenges assessing the conceptual understanding of students with disabilities in statistics. Learn. Disabil. Q. 42, 175–185. doi: 10.1177/0731948718817222

[ref11] Driscoll R.. (2007). Westside test anxiety scale validation (Report No. ED495968). Available online at: https://files.eric.ed.gov/fulltext/ED495968.pdf (Accessed June 29, 2025).

[ref12] Farmer C. Kaat A. J. Edwards M. C. Lecavalier L. (2024). Measurement invariance in intellectual and developmental disability research. Am. J. Intellect. Dev. Disabil. 129, 191–198. doi: 10.1352/1944-7558-129.3.191, 38657963 PMC11095410

[ref13] Finnerty M. S. Jackson L. Ostergren R. (2019). Adaptations in general education classrooms for students with severe disabilities: access, progress assessment, and sustained use. Res. Pract. Pers. Severe Disabil. 44, 87–102. doi: 10.1177/1540796919846424

[ref14] Fornell C. Larcker D. F. (1981). Evaluating structural equation models with unobservable variables and measurement error. J. Mark. Res. 18, 39–50. doi: 10.2307/3151312

[ref15] Frey-Clark M. Natesan P. O’Bryant M. (2019). Assessing statistical anxiety among online and traditional students. Front. Psychol. 10:1440. doi: 10.3389/fpsyg.2019.01440, 31333527 PMC6621917

[ref16] Gallucci M.. (2020). jAMM: jamovi Advanced Mediation Models. [jamovi module]. Available online at: https://jamovi-amm.github.io (Accessed June 4, 2025).

[ref17] Griful-Freixenet J. Struyven K. Verstichele M. Andries C. (2017). Higher education students with disabilities speaking out: perceived barriers and opportunities of the universal Design for Learning framework. Disabil. Soc. 32, 1627–1649. doi: 10.1080/09687599.2017.1365695

[ref18] Grunschel C. Patrzek J. Fries S. (2013). Exploring reasons and consequences of academic procrastination: an interview study. Eur. J. Psychol. Educ. 28, 841–861. doi: 10.1007/s10212-012-0143-4

[ref19] Haryono S. (2017). SEM Method for Management Research with AMOS LISREL PLS. Jakarta: Luxima Metro Media.

[ref20] Hayes A. F. (2022). Introduction to Mediation, Moderation, and Conditional Process Analysis: A Regression-Based Approach. New York: The Guilford Press.

[ref21] Hooper D. Coughlan J. Mullen M. (2008). Structural equation modelling: guidelines for determining model fit. Electron. J. Bus. Res. Methods 6, 53–60. doi: 10.21427/D7CF7R

[ref22] Horlin C. Hronska B. Nordmann E. (2024). I can be a “normal” student: the role of lecture capture in supporting disabled and neurodivergent students’ participation in higher education. High. Educ. 88, 2075–2092. doi: 10.1007/s10734-024-01201-5

[ref23] Jiang X. Goh T. Chen X. Liu M. Yang B. (2023). Investigating university students’ online proctoring acceptance during covid-19: an extension of the technology acceptance model. Australas. J. Educ. Technol. 39, 47–64. doi: 10.14742/ajet.8121

[ref24] Kooren N. S. Van Nooijen C. Paas F. (2024). The influence of active and passive procrastination on academic performance: a meta-analysis. Educ. Sci. 14:323. doi: 10.3390/educsci14030323

[ref25] Kotera Y. Chircop J. Hutchinson L. Rhodes C. Green P. Jones R. . (2021). Loneliness in online students with disabilities: qualitative investigation for experience, understanding and solutions. Int. J. Educ. Technol. High. Educ. 18:64. doi: 10.1186/s41239-021-00301-x, 34909461 PMC8660147

[ref26] Krispenz A. Gort C. Schültke L. Dickhäuser O. (2019). How to reduce test anxiety and academic procrastination through inquiry of cognitive appraisals: a pilot study investigating the role of academic self-efficacy. Front. Psychol. 10:1917. doi: 10.3389/fpsyg.2019.01917, 31481918 PMC6710437

[ref27] Lombardi A. R. Murray C. Gerdes H. (2011). College faculty and inclusive instruction: self-reported attitudes and actions pertaining to universal design. J. Divers. High. Educ. 4, 250–261. doi: 10.1037/a0024961

[ref28] Lomellini A. Lowenthal P. R. Snelson C. Trespalacios J. H. (2025). Accessible and inclusive online learning in higher education: a review of the literature. J. Comput. High. Educ. 37, 1306–1329. doi: 10.1007/s12528-024-09424-2

[ref29] Mastrokoukou S. Kaliris A. Longobardi C. (2025). Drivers of academic procrastination and achievement: a moderated mediation analysis in students with and without specific learning disabilities. J. Learn. Disabil. doi: 10.1177/00222194251391831, 41268788

[ref30] McCloskey J. D. (2011). Academic procrastination scale--short form (APS-S) [database record]. APA PsycTests. doi: 10.1037/t89206-000

[ref31] Mutanga O. (2017). Students with disabilities’ experience in south African higher education – a synthesis of literature. S. Afr. J. High. Educ. 31, 135–154. doi: 10.20853/31-1-1596

[ref32] Núñez-Peña M. I. Bono R. (2020). Math anxiety and perfectionistic concerns in multiple-choice assessment. Assess. Eval. High. Educ. 46, 865–878. doi: 10.1080/02602938.2020.1836120

[ref33] Olson A. J. Leko M. M. Roberts C. A. (2016). Providing students with severe disabilities access to the general education curriculum. Res. Pract. Pers. Severe. Disabil. 41, 143–157. doi: 10.1177/1540796916651975

[ref34] Page A. Charteris J. Anderson J. Boyle C. (2021). Fostering school connectedness online for students with diverse learning needs: inclusive education in Australia during the covid-19 pandemic. Eur. J. Spec. Needs Educ. 36, 142–156. doi: 10.1080/08856257.2021.1872842

[ref35] Panayides P. Papanastasiou E. C. Georgiou K. Karekla M. (2024). Validation of the online test anxiety inventory (on-tai) for adult students. The Rasch measurement approach. Eur. J. Educ. 59:e12724. doi: 10.1111/ejed.12724

[ref36] Park K. So H. Cha H. (2019). Digital equity and accessible moocs: accessibility evaluations of mobile moocs for learners with visual impairments. Australas. J. Educ. Technol. 35, 48–63. doi: 10.14742/ajet.5521

[ref37] Pekrun R. (2006). The control-value theory of achievement emotions: assumptions, corollaries, and implications for educational research and practice. Educ. Psychol. Rev. 18, 315–341. doi: 10.1007/s10648-006-9029-9

[ref38] Pekrun R. (2024). Control-value theory: from achievement emotion to a general theory of human emotions. Educ. Psychol. Rev. 36:83. doi: 10.1007/s10648-024-09909-7

[ref39] Preacher K. J. Hayes A. F. (2008). Asymptotic and resampling strategies for assessing and comparing indirect effects in multiple mediator models. Behav. Res. Methods 40, 879–891. doi: 10.3758/BRM.40.3.879, 18697684

[ref40] Raghunath R. Anker C. Nortcliffe A. (2016). Are academics ready for smart learning? Br. J. Educ. Technol. 49, 182–197. doi: 10.1111/bjet.12532

[ref41] Saleem M. Arsal M. Iqbal U., & others. (2024). Technostress in students and quality of online learning: role of instructor and university support. Front. Educ., 8,:1309642. Doi: 10.3389/feduc.2024.1309642

[ref42] Sarid M. Lipka O. (2023). Students with learning disabilities/attention-deficit/hyperactivity disorder in higher education dealing with remote learning: lessons learned from COVID-19 era. Front. Psychol. 14:1172771. doi: 10.3389/fpsyg.2023.1172771, 37251025 PMC10219608

[ref43] Seale J. (2013). When digital capital is not enough: reconsidering the digital lives of disabled university students. Learn. Media Technol. 38, 256–269. doi: 10.1080/17439884.2012.670644

[ref44] Seale J. (2022). It's not all doom and gloom: what the pandemic has taught us about digitally inclusive practices that support people with learning disabilities to access and use technologies. Br. J. Learn. Disabil. 51, 218–228. doi: 10.1111/bld.12497

[ref45] Steel P. (2007). The nature of procrastination: a meta-analytic and theoretical review of quintessential self-regulatory failure. Psychol. Bull. 133, 65–94. doi: 10.1037/0033-2909.133.1.65, 17201571

[ref46] Steinmayr R. Crede J. McElvany N. Wirthwein L. (2016). Subjective well-being, test anxiety, academic achievement: testing for reciprocal effects. Front. Psychol. 6:1994. doi: 10.3389/fpsyg.2015.01994, 26779096 PMC4705295

[ref47] Svartdal F. Dahl T. I. Gamst-Klaussen T. Koppenborg M. Klingsieck K. B. (2020). How study environments foster academic procrastination: overview and recommendations. Front. Psychol. 11:540910. doi: 10.3389/fpsyg.2020.540910, 33224046 PMC7667251

[ref48] Sweller J. (1988). Cognitive load during problem solving: effects on learning. Cogn. Sci. 12, 257–285. doi: 10.1207/s15516709cog1202_4

[ref49] Tabachnick B. G. Fidell L. S. (2013). Using Multivariate Statistics. 6th Edn Boston: Pearson.

[ref50] Totan T. Yavuz Y. (2009). Westside Sınav Kaygısı Ölçeğinin Türkçe formunun geçerlik ve güvenirlik çalışması. Mehmet Akif Ersoy Üniversitesi Eğitim Fakültesi Dergisi 17, 95–109.

[ref51] United Nations (2015). Transforming our World: The 2030 Agenda for Sustainable Development. New York: United Nations.

[ref52] Woldeab D. Brothen T. (2019). 21st century assessment: online proctoring, test anxiety, and student performance. Int. J. E-Learn. Distance Educ. 34, 1–10.

[ref53] Ye Z. Chi S. Ma X. Pan L. (2025). The impact of basic psychological needs on academic procrastination: the sequential mediating role of anxiety and self-control. Front. Psychol. 16:1576619. doi: 10.3389/fpsyg.2025.1576619, 40463307 PMC12129888

[ref54] Zeidner M. (2005). Test Anxiety: The state of the Art. New York: Kluwer Academic Publishers.

[ref55] Zimmerman B. J. (2000). “Attaining self-regulation: a social cognitive perspective,” in Handbook of Self-Regulation, eds. Boekaerts M. Pintrich P. R. Zeidner M. (San Diego: Academic Press), 13–39.

